# Insight into the Influence of the Chiral Molecular Symmetry on the Chiroptics of Fluorescent BINOL-Based Boron Chelates ^†^

**DOI:** 10.3390/ecsoc-24-08308

**Published:** 2020-11-14

**Authors:** Josué Jiménez, Edurne Avellanal-Zaballa, Sergio Serrano, Alexis R. Torres, Antonia R. Agarrabeitia, Florencio Moreno, Gilles Muller, Jorge Bañuelos, Beatriz L. Maroto, Santiago de la Moya

**Affiliations:** 1 Departamento de Química Orgánica, Facultad de Ciencias Químicas, Universidad Complutense de Madrid, Ciudad Universitaria s/n, 28040 Madrid, Spain; 2 Departamento de Química Física, Facultad de Ciencia y Tecnología, Universidad del País Vasco-EHU, 48080 Bilbao, Spain; 3 Department of Chemistry, San José State University, San José, CA 95192-0101, USA

**Keywords:** chiroptics, boron chelates, organic dyes, chiral molecular symmetry, CPL

## Abstract

The prominent influence of the molecular symmetry, as defined by the symmetry point group, on the chiroptical behavior (electronic circular dichroism and, especially, circularly polarized luminescence) of simple fluorescent boron chelates (BODIPY and related BOPHY analogues) is studied and discussed. It is shown that increasing the dye symmetry by means of the *D_3_* chiral symmetry group is a workable design option to enhance the level of differential emission of right- and left-circularly polarized light in BODIPY dyes and related emitters, and that the influence of the level of symmetry is stronger than the influence of the higher number of chiral moieties perturbing the acting achiral chromophore.

## 1. Introduction

Among all the technological applications based on photonics, those based on chiroptical phenomena (particularly, circularly polarized luminescence, CPL) have special relevance, mainly owing to the higher resolution of the circularly polarized over the linearly polarized or the unpolarized light. The higher resolution is due to the existence of two additional parameters: level and handedness of the circular polarization. Therefore, CPL-enabling systems have a huge potential for the development of advanced photonic materials for devices based on chiral properties, enantioselective sensing systems, biomedical applications, 3D displays, OLED materials, optoelectronic devices, spintronics devices, security inks, information storage, or smarter chiroptical switching devices [1-4].

**Figure 1.**
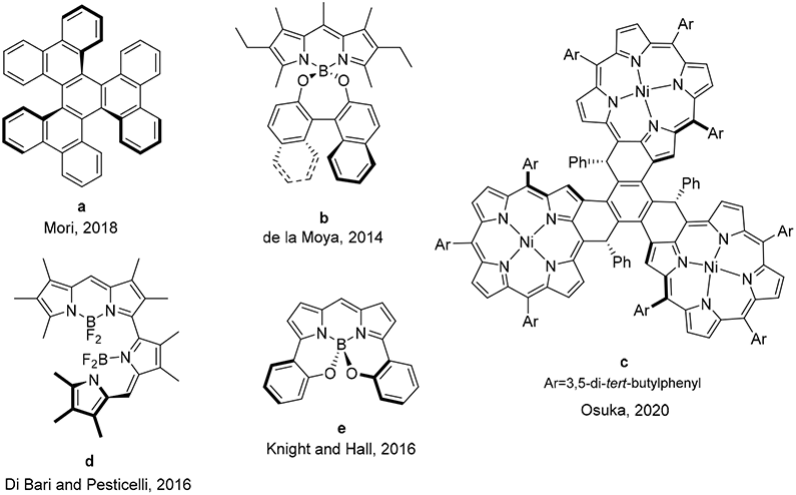
Some examples of CPL-SOMs [10-14].

However, the design of emitters efficiently enabling CPL is not an easy task because it is necessary to combine, in the same structure, a high fluorescence quantum yield and a high level of circular polarization in the emission [5]. Among all the possible chemical systems that show circularly polarized luminescence, CPL-SOMs (simple organic molecules enabling CPL, see some examples in Figure 1) have gained exponential interest in the last years. The reason for the growing interest in these emitters lies in their potential for the development of specific CPL materials due to properties associated with their organic nature, such as low molecular weight or organic-solvent solubility, and their high capacity of absorbing and emitting light. Additionally, the photophysical signatures of SOMs can be adjusted by workable chemical modifications of the chromophore, which provides added value to this kind of emitters [6-9].

The level of CPL is quantified by the luminescence dissymmetry factor, *g*_lum_, which is defined by the difference emission divided by the averaged emission intensity, that is, *g*_lum_ = (*λ*) = 2Δ*I*/*I* = 2(*I*_L_ − *I*_R_)/(*I*_L_ + *I*_R_), where *I*_L_ and *I*_R_ refer, respectively, to the intensity of left and right circularly polarized emissions. The values of *g*_lum_ are found between −2 and +2 (completely right- and left-polarized emission, respectively). As CPL-emitters, organic chromophores have the advantage of showing high emission (high fluorescence quantum yields). However, their levels of circular polarization reported to date are still very small (*g*_lum_ values are typically in an order of 10^−3^ to 10^−5^) [5].

Molecular symmetry plays an important role in the CPL response (see, for example, *D*_3_-symmetric triple pentahelicene **a** [10], *C*_2_-symmetric BODIPYs **b** [11], **d** [13] and **e** [14], or *C*_3_-symmetric porphyrin trimer **c** [12], in Figure 1), owing to its influence on the electric and magnetic transition dipole moments of the molecule involved in the circularly polarized emission [15]. However, the role of molecular symmetry in the CPL emission is not completely known yet. In this regard, according to our knowledge, there are no systematic studies directed towards a deeper knowledge on the relationship between the chiral molecular symmetry and the chiroptical behavior of CPL-SOMs. This kind of study would contribute to the rational design of CPL-SOMs for advanced photonic applications.

**Figure 2.**
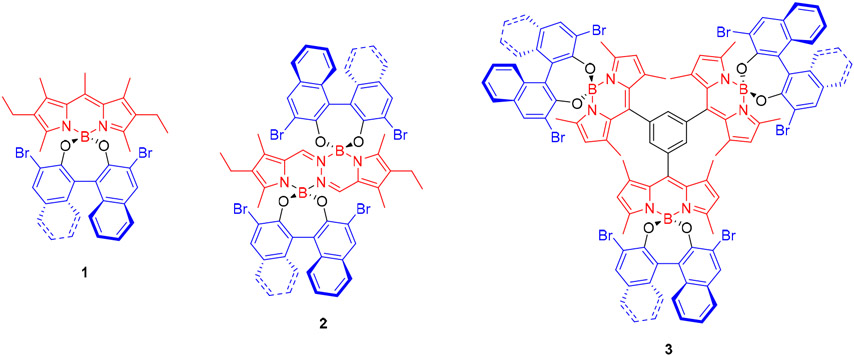
CPL-SOMs based on BODIPY (**1** and **3**) or BODIPY analogue (BOPHY, **2**) selected for this study.

In this scenario, we decided to study the CPL behavior of three molecular dyes based on BODIPY (BOron DIPYrromethene) or BOPHY (hydrazine-derived BODIPY analogue, see Figure 2) and relate this behavior with the molecular chirality (level of molecular symmetry and ratio of chiral perturbing units *per* chromophoric unit) of the dyes.

## 2. Results and Discussion

For our study, we selected the CPL-SOMs shown in Figure 2. All these molecules are based on BODIPY or BOPHY as the absorbing and emitting chromophore and (*R*)-3,3′-dibromoBINOL (3,3′-dibromo-1,1′-bi(naphth-2-ol)) as the chiral unit, but they have different molecular symmetry (chiral symmetry point group) and/or a different number of chiral BINOL units. The three of them are based on the new structural design recently reported by us to achieve CPL from SOMs, where the only role of the BINOL moiety is to chirally perturb the inherently achiral BODIPY (or BOPHY) chromophore [11].

BODIPY dye **1** (Figure 2) is a *C*_2_-symmetric molecule, where the *C*_2_ symmetry comes from a single chiral BINOL unit. The structure of BOPHY dye **2** (Figure 2) keeps the *C*_2_-symmetry of **1**, but duplicates the number of BINOL moieties. Dyes **1** [16] and **2** [17] have been reported before by us and show maximum *g*_lum_ values of −0.6 × 10^−3^ and −1.4 × 10^−3^, respectively, in the visible region upon visible irradiation (Table 1). These *g*_lum_ values are in the typical range for CPL-SOMs [7]. Noticeably, the ∣*g*_lum_∣ value of **2** is 2.3 times higher than that of **1**. This significant increase of the level of circular polarization could be explained by the duplicated number of chiral moieties perturbing the acting chromophore. Strikingly, this is not the trend observed in circular dichroism. Thus, **2** has a maximum absorption dissymmetry factor, *g*_abs_ [18], in the visible region that is slightly smaller, in absolute value, than that exhibited by **1** under similar conditions (see Table 1). This fact reveals an additional possible key factor affecting the chiroptical behavior of both dyes: the nature of the acting chromophore (i.e., BODIPY vs. BOPHY).

To get a deeper insight on the possible beneficial influence of symmetry vs. number of chiral perturbing moieties per chromophoric unit on the CPL activity of these boron-chelate dyes, we decided to synthesize **3**, which is based on a BODIPY trimer with pendant chiral BINOL units, and study its CPL behavior. The symmetry (i.e., number of symmetry elements) of **3** is higher than in **1** or **2**: dye **3** is *D*_3_-symmetric, whereas **1** and **2** are *C*_2_-symmetric. However, the number of perturbing chiral moieties per chromophoric unit is only one, as in the case of **1**; as well as the type of acting chromophore (BODIPY in both cases).

**Scheme 1.**
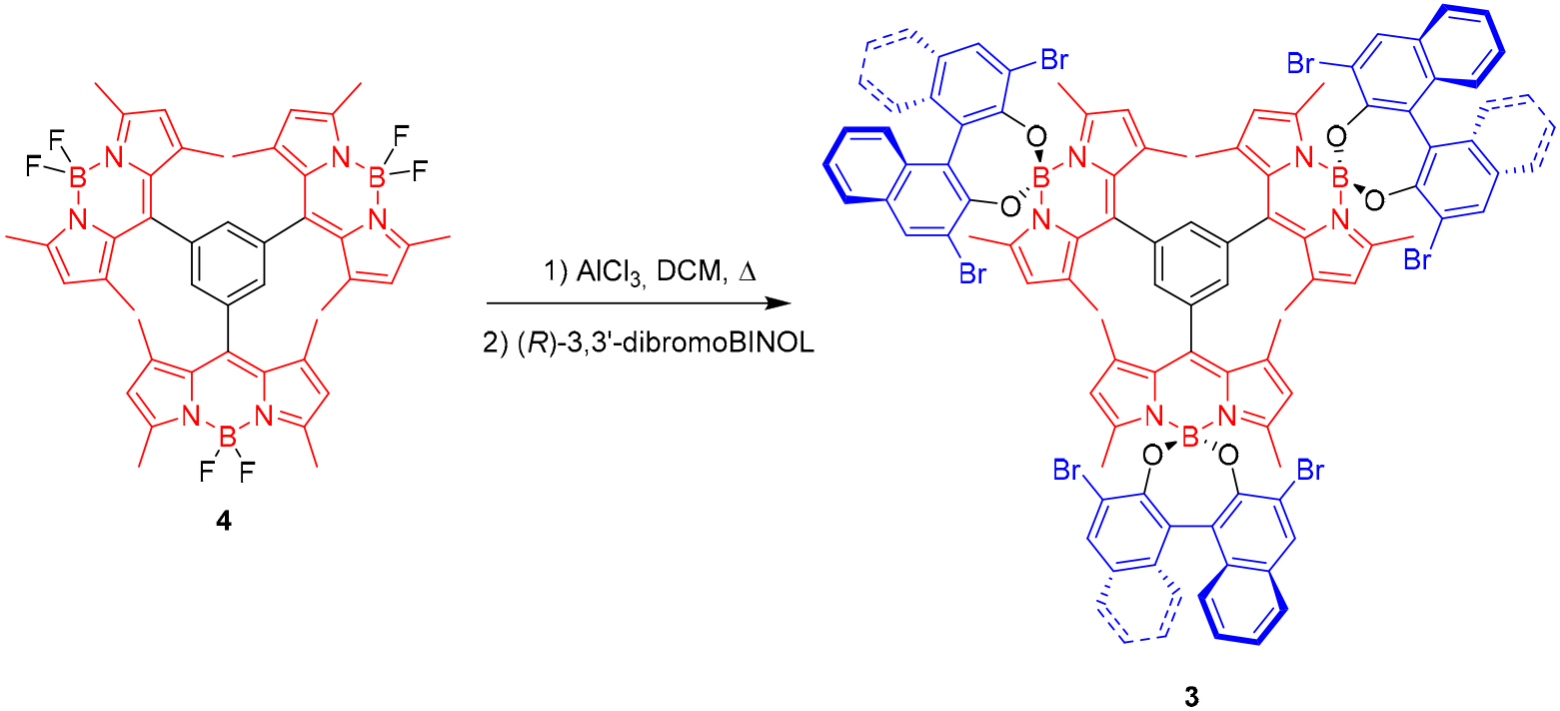
Synthesis of *D*_3_-symmetric **3** from its parent *F*-BODIPY trirner. BINOL: 1,1′-bi(napht-2-ol).

Dye **3** was synthesized by nucleophilic substitution of fluorine in parent *F*-BODIPY **4** [19], activated by aluminum trichloride (Scheme 1). Starting trimeric *F*-BODIPY was prepared from 2,4-dimethylpyrrole and benzene-1,3,5-tricarbonyl trichloride, according to a previously described procedure [19].

**Figure 3.**
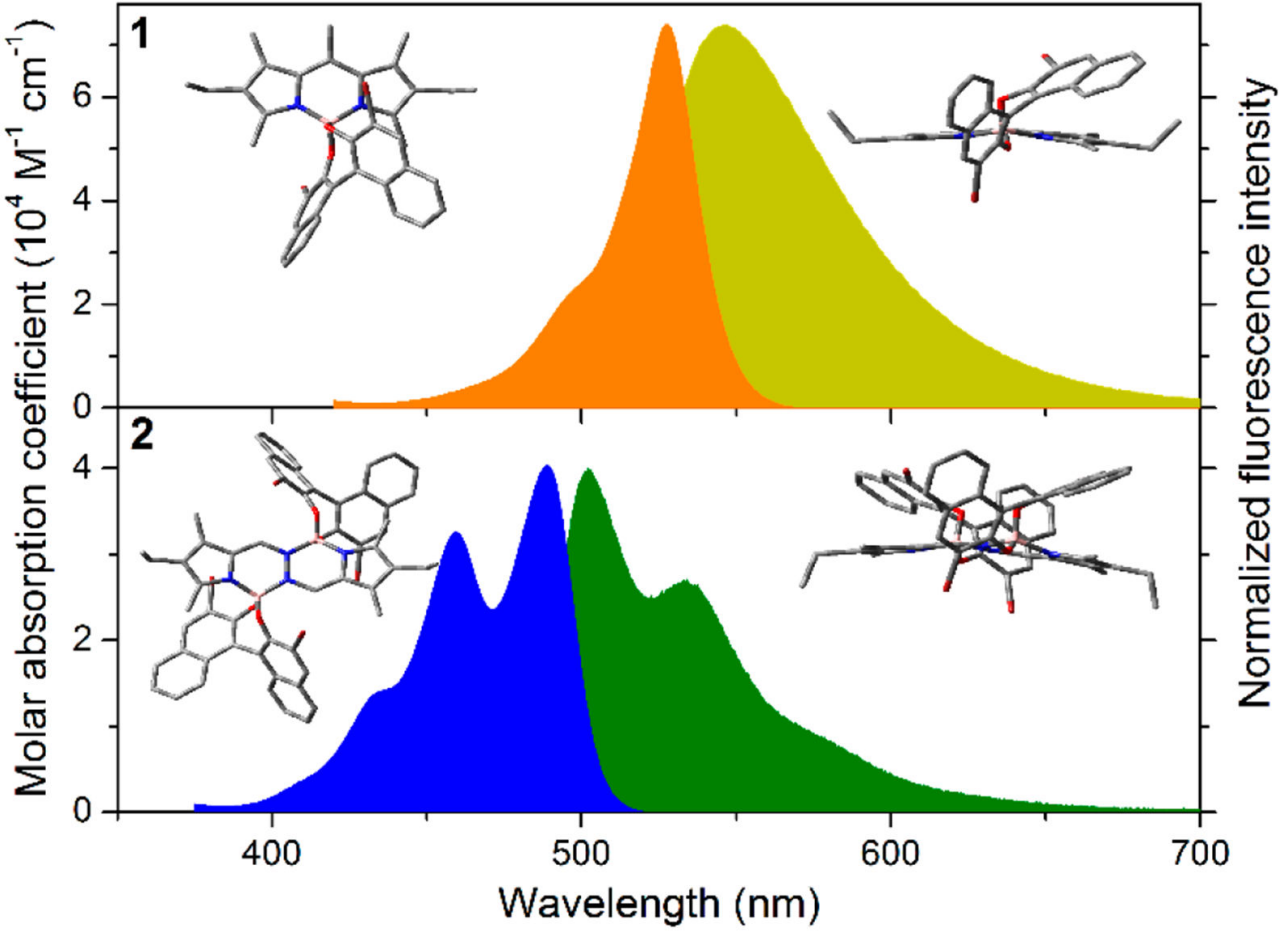
Visible absorption spectra and normalized visible fluorescence spectra of BODIPY-based dye **1** and BOPHY-based dye **2** in diluted solution of chloroform, and corresponding optimized ground state geometries (b3lyp/6-31+g*) in chloroform (PCM) in frontal- (left) and side-views (from the boron atom, right).

The profiles of the spectral bands recorded for the bromoBINOLated dyes **1** and **2** fully remain to their respective *F*-BODIPY and *F*-BOPHY counterparts (Figure 3), supporting the low impact of the chiral unit into the chromophoric delocalized *π*-systems [16,17]. In spite of the more extended *π*-system of the BOPHY derivative **2**, its spectral bands feature a higher vibrational resolution and are hipsochromically shifted with regard to the BODIPY-based dye **1**. The computationally optimized geometries reveal that the dipyrrin framework of compound **1** is slightly bended to accommodate the bromoBINOL at the boron center. However, the bending of the chromophoric core is more prominent in compound **2**, with each boron-pyrromethene placed in different planes [17]. Surely, such planarity distortion decreases the chromophore *π*-con]ugation in **2**, explaining the observed hypsochromic shift in both bands, as well as the lower absorption capability with regards to dye **1** [17] (Figure 3).

Both dyes display reasonable high fluorescence emissions (Table 2). The key role of the bromine atoms decorating the BINOL moiety is noteworthy. It is known that the electron donating ability of the BINOL is able to induce intramolecular charge transfer (ICT) processes that quench the fluorescence response of both BODIPY and BOPHY [16,17]. Thanks to the electronegativity of this heteroatom, bromination is a suitable strategy to make the BINOL less prone to induce ICT, restoring the expected bright emission from both chromophores. The BODIPY-based dye **1** is more fluorescent than the BOPHY-based dye **2** (69% vs. 48%, respectively in Table 2). Such a trend is likely related to the aforementioned distortion of planarity in the latter compound, which enhances the probability of non-radiative deactivation pathways, as supported by the faster lifetimes (from 6.56 ns in dye **1** to 2.00 ns in dye **2**, Table 2) [17].

The profile and position of the absorption and fluorescence bands of the multichromophoric dye **3** fully remains to that of dye **1** (Table 2). Such a feature suggests that the imposed steric hindrance through alkylation at the neighboring 1 and 7 positions around the *meso* one avoids electron coupling between the electronic clouds of the BODIPY cores and the central benzene scaffold. However, the intensity of both spectral bands is much lower than expected. The simultaneous presence of up to three chromophoric subunits should give rise to a much stronger absorption band if each BODIPY contributes additively to the whole absorption band. Besides, compound **3** is poorly fluorescence (Table 2), in spite of the restricted conformational freedom, which hinders non-radiative relaxations related with internal conversion, and the feasibility of excitation energy migration between the chromophoric subunits owing to the short intramolecular distance imposed by the covalent linkage. However, such spatial proximity enables ICT processes between the BODIPYs that comprise the assembly **3**. Indeed, ICT processes have been reported in the literature for symmetrical covalently linked dimers, as consequence of a symmetry breaking charge transfer (SBCT) mechanism, which takes place between an identical pair of chromophores close enough arranged [20]. Therefore, the ongoing ICT rules the photophysics of triad 3, explaining its almost negligible emission (just 4%, Table 2) and the fast dynamics at the excited state (less than 400 ps, Table 2). Moreover, the ICT is so stabilized, due to the presence of three identical units, that even the absorption acquires a CT character, explaining why the absorption is not the sum of each individual chromophore.

Next, the chiroptical behavior of new *D*_3_-symmetric **3** was studied, in comparison with *C*_2_-symmetric **1** and **2**. The CD and CPL activity of **3** were measured from chloroform solutions (ca. 10^−6^ M for CD and ca. 10^−3^ M for CPL) and the corresponding maximum *g*_abs_ and *g*_lum_ values were calculated from the recorded visible CD or CPL spectra, respectively (see Table 1). Interestingly, both the ∣*g*_abs_∣ and the ∣*g*_lum_∣ values for *D*_3_-symmetric **3** are higher than those for *C*_2_-symmetric **1** or **2**. Indeed, ∣*g*_abs_∣ for **3** is up to 3.5 times higher than ∣*g*_abs_∣ for **1** or **2** and ∣*g*_lum_∣ for **3** is up to 3.3 times higher than those for **1** or **2**. These results confirm that the molecular symmetry is a key structural feature to take into account when designing BODIPY-based (or BODIPY-like) dyes with chiroptical properties. The results also support the interest of the highly symmetric *D*_3_ chiral symmetry point group for the development of CPL-SOMs.

## 3. Conclusions

The results reported herein show that the enhancement of the molecular symmetry serves better to the consecution of higher levels of circular polarization in BODIPY-based (or BODIPY like) CPL-SOMs than the increase of the number of chiral moieties perturbing the involved acting chromophore (ratio of chiral units per achiral chromophore). This fact should be the same for emitters based on other chromophores. In this regard, the *D*_3_ symmetry, up to date poorly described in CPL-SOMs when compared to *C*_1_ and *C*_2_ symmetries, should be a valuable option to take into account when designing emitters of circularly polarized light for technological applications.

## 4. Materials and Methods

### 4.1. Synthesis

#### General.

Common solvents were dried and distilled by standard procedures. All starting materials and reagents were obtained commercially and used without further purifications. Elution flash chromatography was conducted on silica gel (230 to 400 mesh ASTM). Thin layer chromatography (TLC) was performed on silica gel plates (silica gel 60 F254, supported on aluminum). The NMR spectra were recorded at 20 °C, and the residual solvent peaks were used as internal standards. The NMR signals are given in ppm. DEPT-135 NMR experiments were used for the assignation of the type of carbon nucleus (C, CH, CH_2_, and CH_3_). The FTIR spectra were recorded from neat samples using the ATR technique and IR bands are given in cm^−1^. Optical rotations in chloroform solution (dye concentration, *c*, expressed in g/100 mL) were recorded at 293 K on an Anton Paar MCP 100 polarimeter.

#### Synthesis of 3:

A mixture of **4** [19] (50 mg, 0.06 mmol) and aluminum chloride (61 mg, 0.46 mmol) in dry CH_2_Cl_2_ (10 mL) was refluxed under argon atmosphere until reaction completion (disappearance of starting material monitored by TLC). The mixture was cooled down to room temperature and, then, a solution of enantiopure (*R*)-(+)-3,3′-dibromo-1,1′-bi(naphth-2-ol) (136 mg, 0.31 mmol) in anhydrous acetonitrile (5 mL) was added dropwise and the resulting mixture was stirred at r.t. for additional 6 h. Then, the reaction mixture was diluted with CH_2_Cl_2_ (10 mL), washed with water (1 × 10 mL) and dried over anhydrous Na_2_SO_4_. After filtration and solvent evaporation under reduced pressure, the obtained residue was purified by flash chromatography (hexane/CH_2_Cl_2_ 7:3) to afford **3**. 15 mg (12%). Red solid. R_*f*_ = 0.35 (hexane/CH_2_Cl_2_ 1:1). $[\alpha {]}_{20}^{D}$ −3227.5 (*c* 0.071 CHCl_3_). ^1^H NMR (CDCl_3_, 300 MHz) *δ* 8.15 (s, 6H), 7.98 (s, 3H), 7.75 (d, *J* = 8.1 Hz, 6H), 7.32 (m, 6H), 7.13 (m, 6H), 7.05 (d, *J* = 8.6 Hz, 6H), 5.92 (s, 6H), 1.88 (s, 18H), 1.74 (s, 18H) ppm. ^13^C NMR (CDCl_3_, 75 MHz) *δ* 157.4 (C), 150.5 (C), 141.8 (C), 136.0 (CH), 139.3 (C), 133.8 (C), 133.2 (C), 133.18 (C), 132.15 (CH), 130.14 (C), 127.2 (CH), 127.1 (CH), 125.9 (CH), 124.6 (CH), 124.4 (CH), 122.5 (C), 119.1 (C), 18.5 (CH_3_), 15.9 (CH_3_) ppm. FTIR *v* 1540, 1502, 1395, 1158, 983 cm^−1^.

### 4.2. Spectroscopic Measurements and Quantum Mechanic Calculations

The photophysical properties were registered using quartz cuvettes with optical pathways of 1 cm in diluted solutions (around 2 × 10 ^−6^ M), prepared by diluting the concentrated stock solution in chloroform. Ultraviolet-visible (UV-vis) absorption and fluorescence spectra were recorded on a Varian model CARY 4E spectrophotometer and an Edinburgh Instruments spectrofluorimeter (model FLSP920), respectively. Fluorescence quantum yields (*φ*) were obtained using PM567 (Exciton, *φ*^r^ = 0.84), Coumarine 152 (Kodak, *φ*^r^ = 0.2), and PM546 (Exciton, *φ*^r^ = 0.85), in ethanol as references for dyes **1**, **2** and **3**, respectively. Radiative decay curves were registered with the time correlated single-photon counting technique, as implemented in the aforementioned spectrofluorimeter. Fluorescence emission was monitored at the maximum emission wavelength, by means of a microchannel plate detector (Hamamatsu C4878) of picosecond time-resolution (20 ps), after excitation with a wavelength-tunable Fianium pulsed laser (time resolution of around 150 picoseconds). The fluorescence lifetime (*τ*) was obtained after the deconvolution of the instrumental response signal from the recorded decay curves by means of an iterative method. The goodness of the exponential fit was controlled by statistical parameters (chi-square) and the analysis of the residuals.

Ground state energy minimizations were performed using hybrid b3lyp functional, within the Density Functional Theory (DFT), using the triple valence basis set with a polarization and diffuse function (6-311+g*). The optimized geometries were taken as a true energy minimum using frequency calculations (no negative frequencies). The Polarizable Continuum Model (PCM) was considered to describe the solvent effect (chloroform) during energy minimizations. All the calculations were performed in Gaussian 16, using the “arina” computational resources provided by the UPV-EHU.

### 4.3. CD and CPL Measurements

CD spectra were recorded on a Jasco (model J-715) spectropolarimeter using standard quartz cells of 1 cm optical-path length in chloroform solution, at a dye concentration of 4.6 × 10^−6^ M. Circularly polarized luminescence (CPL) and total luminescence spectra were recorded at 295 K in degassed c-hexane solution at a dye concentration of ca. 2 mM, on an instrument described previously [21], operating in a differential photon-counting mode. The light source for excitation was a continuous wave 1000 W xenon arc lamp from a Spex Fluorolog-2 spectrofluorimeter, equipped with excitation and emission monochromators with dispersion of 4 nm/mm (SPEX, 1681B). To prevent artefacts associated with the presence of linear polarization in the emission [22], a high-quality linear polarizer was placed in the sample compartment and aligned so that the excitation beam was linearly polarized in the direction of emission detection (*z*-axis). The key feature of this geometry is that it ensures that the molecules that have been excited and that are subsequently emitting are isotropically distributed in the plane (*x,y*) perpendicular to the direction of emission detection. The optical system detection consisted of a focusing lens, a long pass filter, and a 0.22 m monochromator. The emitted light was detected by a cooled EMI-9558B photomultiplier tube operating in photo-counting mode.

## Figures and Tables

**Table 1.** Maximum *g*_lum_ (chromophore visible excitation) and *g*_abs_ (chromophore visible absorption) of dyes **1, 2** and **3** in chloroform solution (micromolar for *g*_abs_; milimolar for *g*_lum_).

Dye	*g*_abs_•10^3^	*g*_lum_•10^3^
**1**	−0.9	−0.6
**2**	−0.4	−1.4
**3**	−1.4	−2.0

**Table 2.** Photophysical properties of dyes **1, 2** and **3** in chloroform solution (micromolar). Absorption wavelength (*λ*_ab_), molar absorption coefficient (*ε*_max_), fluorescence wavelength (*λ*_fl_), quantum yield (*φ*) and lifetime (*τ*).

Dye	*λ*_ab_ (nm)	*ε_max_* (10^4^ M^−1^ cm^−1^)	*λ*_fl_ (nm)	*φ*	*τ* (ns)
**1**	527.0	7.4	547.0	0.69	6.56
**2**	486.0	4.0	501.0	0.48	2.00
**3**	519.0	10.5	551.0	0.04	0.37 (93%)/2.31 (7%)
